# Early prediction of final body weight in Hanwoo steers using machine and deep learning models

**DOI:** 10.5713/ab.250595

**Published:** 2025-11-10

**Authors:** Eunjeong Jeon, Joonpyo Oh

**Affiliations:** 1 Department of Animal Science, College of Agriculture and Natural Resources, Michigan State University, East Lansing, MI, USA; 2 Department of International Agricultural Technology, Graduate School of International Agricultural Technology, Seoul National University, Pyeongchang, Korea

**Keywords:** eXtreme Gradient Boosting, Final Body Weight, Hanwoo Steers, Long Short-term Memory, Precision Livestock Farming, Random Forest

## Abstract

**Objective:**

Accurate early prediction of final body weight (BW) is essential for optimizing feeding strategies and slaughter planning in beef cattle production. This study compared the performance of three machine learning models (k-nearest neighbors, random forest, and eXtreme Gradient Boosting) and one deep learning model (long short-term memory [LSTM]) to forecast the final BW of Hanwoo steers at various time points prior to slaughter.

**Methods:**

A total of 196 Hanwoo steers (7 to 31 months of age) from a commercial farm were utilized. Input data included monthly BW and feed nutrient intake (crude protein, ether extract, neutral detergent fiber, and total digestible nutrients) across three growth stages. Six input configurations (I1–I6) were designed to predict the final BW at 17, 13, 9, 6, 3, and 1 month(s) before slaughter, with a target age of 31 months. The machine and deep learning models were assessed by five-fold cross-validation (training set) and a test set and evaluated via the coefficient of determination (R^2^) and root mean squared error (RMSE).

**Results:**

Among the tested models, the LSTM achieved the highest prediction accuracy across all the configurations. The performance of the LSTM improved as the prediction point approached the target slaughter age: I1 (R^2^ = 0.60, RMSE = 52.80), I2 (0.72, 45.40), I3 (0.76, 40.92), I4 (0.83, 35.84), I5 (0.90, 33.12), and I6 (0.97, 22.62).

**Conclusion:**

These results demonstrated that LSTM effectively captured temporal dependencies in sequential data, enabling more accurate BW forecasting under commercial conditions. While I6 achieved the highest prediction accuracy, the 3–6 month predictions (I4 and I5) demonstrated reasonably high accuracy, which could provide a practical timeframe for farm-level management and planning. This approach could be used in evidence-based decision-making in Hanwoo production by providing reliable predictions well before slaughter.

## INTRODUCTION

Body weight (BW) is widely recognized as a fundamental indicator in cattle production, contributing to the evaluation of growth performance, productivity, health conditions, and nutritional strategies across different growth stages [1–3]. To ensure optimal productivity and sustain farm profitability, livestock producers are required to regularly monitor the status of individual animals and make timely and informed decisions [4,5]. Among these decisions, early estimation of the target BW can play a pivotal role in farm management, as it is closely linked to slaughter timing and economic outcomes [2,6,7]. Delayed slaughter beyond the target BW may result in unnecessary feed costs and ultimately impair efficient resource use [8].

In recent years, the livestock industry has shown increasing interest in leveraging historical data to enhance the current productivity and decision-making [9–11]. However, in large-scale commercial operations, BW data are often difficult to collect on a regular basis because of labor constraints, equipment costs, and potential stress to animals during handling [2,5]. Even when BW records are available, such data are frequently underutilized because of the limited accessibility to appropriate analytical tools and predictive frameworks. As a result, producers are prone to relying on visual judgment, intuition, and experience-based decisions rather than evidence-based strategies. To address these limitations, predictive tools that leverage historical data offer promising approaches for improving the efficiency of beef production [12].

BW has primarily been estimated indirectly by morphometric traits with techniques ranging from simple tools such as measuring tapes [13] to advanced technologies that extract features from devices such as two-dimensional (2D) or three-dimensional (3D) cameras, ultrasound, and infrared sensors [2,14,15]. In the case of Hanwoo cattle, several studies have proposed models to estimate BW via body size measurements [16] or 3D imaging techniques [17]. To the best of our knowledge, the application of machine learning (ML) and deep learning (DL) models to predict the final BW represents a novel approach in Hanwoo steers, as previous studies have mainly focused on estimating current BW using morphometric or imaging-based methods. Using ML and DL models with sequential feeding and BW data enables time-ahead prediction of final BW without relying on specialized equipment.

A range of supervised ML algorithms such as k-nearest neighbors (KNN), random forest (RF), and eXtreme Gradient Boosting (XGBoost) have been widely applied to predict weight-related traits in beef cattle because of their ability to model complex nonlinear relationships in structured tabular data [5,16]. While conventional ML models such as KNN, RF and XGBoost often perform well on static structured datasets by effectively learning nonlinear interactions among input variables, they do not inherently model temporal dependencies in sequential data. The long short-term memory (LSTM) network is a DL model specifically designed to handle sequential data, effectively capturing both short- and long-term temporal dependencies [18,19]. Its strengths have been demonstrated across various fields including engineering [20,21], medicine [22], and animal science [23,24]. In the context of Hanwoo production, LSTM may provide a useful framework for modeling individual growth trajectories using historical data.

Therefore, this study aims to compare the prediction performance of both the ML and DL models for forecasting the final BW of Hanwoo steers at different time points prior to slaughter. By incorporating historical monthly BW and feed nutrient intake data, we hypothesize that the LSTM model will yield superior prediction accuracy due to its temporal modeling capacity.

## MATERIALS AND METHODS

### Data collection

The data used in this study were collected from eight different feeding experiments conducted at the Cargill Technology Application Center between October 2018 and November 2023 located in Daehwa-myeon, Pyeongchang-gun, South Korea. A total of 196 Hanwoo steers (206.35±27.71 kg BW on average at the beginning of the experiments) were used in the trials. BW was measured monthly from 7 to 31 months of age using a scale in the morning prior to the first feeding. The animals were housed in an open and well-ventilated barn under standard farm management practices, with four steers allocated to a pen.

The feeding period of the animals was divided into three stages: growing (7–14 months), early fattening (15–22 months), and late fattening (23–31 months of age). All the experimental diets were sampled at the beginning of each of the eight feeding experiments conducted during the study period. The chemical compositions of the samples were analyzed according to standard methods of the Association of Official Analytical Chemists [25]. The total digestible nutrient (TDN) values were estimated following the National Research Council guidelines [26]. The detailed chemical compositions of the diets used at each stage were provided in Table 1.

It should be noted that feed intake for each pen was daily monitored only during the first week of each month, and the average daily intake for that week was used as daily feed intake for the month. Although this approach was necessary for practical reasons, it might not fully capture within-month variation in feed intake, which could result in some estimation bias. Amount of nutrient intake was calculated based on feed intake and feed composition by the following equations:


(1) CP intake (CPI)=Feed intake (kg/d, DM basis)×CP (%)/100

(2) EE intake (EEI)=Feed intake (kg/d, DM basis)×EE (%)/100

(3) NDF intake (NDFI)=Feed intake (kg/d, DM basis)×NDF (%)100

(4) TDN intake (TDNI)=Feed intake (kg/d, DM basis)×TDN (%)/100

where DM represents dry matter; CP, crude protein; EE, ether extract; NDF, neutral detergent fiber; and TDN, total digestible nutrient. The descriptive statistics of all the variables used in the analysis are presented in Table 2.

### Model development

**Figure 1:**
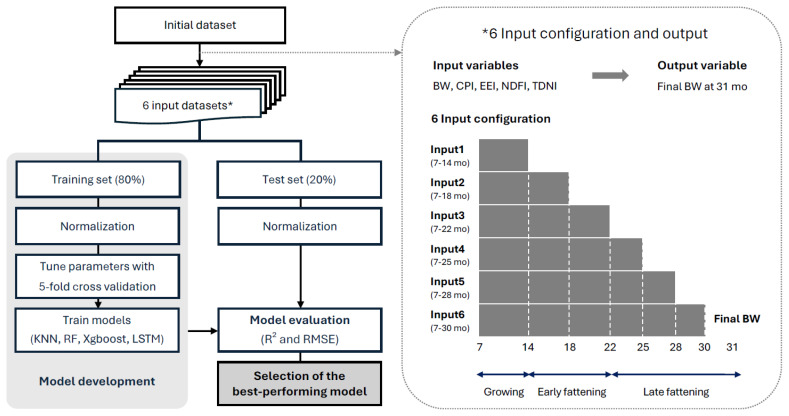
Workflow for model development and evaluation based on six input configurations varying in temporal length for predicting the final body weight (BW). Growth stages were defined as follows: growing (7–14 months), early fattening (15–22 months), and late fattening (23–31 months). All input configurations used five variables: BW, crude protein intake (CPI), ether extract intake (EEI), neutral detergent fiber intake (NDFI), and total digestible nutrient intake (TDNI). KNN, k-nearest neighbors; RF, random forest; XGBoost, eXtreme Gradient Boosting; LSTM, long short-term memory; R^2^, coefficient of determination; RMSE, root mean square error.

We designed six input configurations (I1–I6) based on varying temporal windows from 7 months of age up to 14, 18, 22, 25, 28, and 30 months corresponding to prediction points 17, 13, 9, 6, 3, and 1 month(s) before slaughter, respectively (Table 3, Figure 1). Each configuration used the same five input variables: BW, crude protein intake (CPI), ether extract intake (EEI), neutral detergent fiber intake (NDFI), and total digestible nutrients intake (TDNI), while differing in input sequence length (from 8 to 24 months) to assess how early the final BW could be accurately predicted. The input structures were visualized as sequential blocks in the model development workflow to clearly represent the temporal aspect of the prediction task. The input configurations were applied consistently across all the models (KNN, RF, XGBoost, and LSTM) to ensure fair comparison. For the LSTM model, the input sequences were structured as three-dimensional arrays (samples×time steps× features), with each time step containing five variables: BW, CPI, EEI, NDFI, and TDNI.

Prior to model training, data preprocessing was performed to ensure data quality and consistency. Of the 196 Hanwoo steers initially recorded, one animal was excluded due to an abnormal BW record identified as an outlier, and 53 animals with missing BW data at any month between 7 and 31 months of age were removed. Consequently, a total of 142 animals were retained for the final analysis. The dataset was split into training (80%) and testing (20%) sets by stratified sampling based on the target BW. Within the training set, 5-fold cross-validation was conducted at the individual animal level, with 80% of the animals used for training and 20% for validation in each fold.

All continuous input and output variables were normalized via min–max scaling prior to model training and evaluation, as shown in the following equation [27]:


(5) $$X_{\text{norm}}=\frac{X-X_{\text{min}}}{X_{\text{max}}-X_{\text{min}}}$$

After prediction, the normalized output values were converted back to their original scale using inverse transformation [27]:


(6) $$\text{Y}={\text{Y}}_{\text{norm}}\times ({\text{Y}}_{\text{max}}-{\text{Y}}_{\text{min}})+{\text{Y}}_{\text{min}}$$

In these equations, X and X_norm_ represent the original and normalized values of the variables, respectively, while X_min_ and X_max_ denote their minimum and maximum values, respectively. Y and Y_norm_ refer to the original and normalized predicted output values, with Y_min_, Y_max_ referring to the minimum and maximum values of the output variable, respectively. This procedure enabled the interpretation of the predicted values in actual weight units (kg), ensuring their practical usability.

### Hyperparameter tuning

To ensure fair model comparison and robust performance, all the models underwent hyperparameter optimization with a grid search approach based on five-fold cross-validation within the training set. The KNN model was implemented using the caret package in R. The number of neighbors (k) was tested at 3, 5, and 7. The configuration with k = 5 yielded the best validation result and was selected as the final model.

The randomforest package in R was used to implement the RF model. Three hyperparameters were tuned: the number of trees was tested at 300, 500, and 700; the number of features randomly selected at each split (mtry) at 2, 3, and 4; and the minimum size of terminal nodes (nodesize) at 3, 5, and 7. The final configuration, which used 500 trees, mtry = 3, and nodesize = 5, provided the best cross-validation performance. The XGBoost model was implemented using the xgboost package in R. The learning rate was tested at 0.01, 0.05, and 0.1; the maximum tree depth at 4, 6, and 8; and the number of boosting rounds at 50, 100, and 150. The combination of a learning rate of 0.1, a maximum depth of 6, and 100 boosting rounds yielded the best performance and was selected as the final model. The keras and tensorflow packages were utilized to develop the LSTM model in R. The grid search explored combinations of the number of LSTM units set to 32, 64, and 96; dropout rates of 0.2, 0.3, and 0.4; learning rates of 0.001, 0.003, and 0.005; batch sizes of 16 and 32; and training epochs of 50 and 100. All the LSTM model combinations were trained with the Adam optimizer and the mean squared error (MSE) as the loss function. The final model consisted of a single LSTM layer with 64 hidden units, followed by a dropout layer (rate = 0.2), and a dense output layer with one unit. It was trained for 100 epochs with a batch size of 32 and a learning rate of 0.005. All the models were evaluated through the same five-fold cross-validation strategy within the training set and tested on an independent test set. This procedure was repeated for each of the six input configurations (I1–I6), allowing consistent performance comparisons across varying temporal input lengths.

### Model evaluation

The average and standard deviation (SD) of two performance metrics were calculated: the coefficient of determination (R^2^) and the root mean squared error (RMSE). Following cross-validation, the models were retrained on the entire training dataset and then evaluated on the test set to assess the generalization performance. The metrics were calculated as follows:


(7) $$R^{2}=1-\frac{\sum {\left(y_{i}-{\hat{y}}_{i}\right)}^{2}}{\sum {\left(y_{i}-\bar{y}\right)}^{2}}$$

(8) $$RMSE=\sqrt{\frac{1}{n}\sum_{i=1}^{n}{\left(y_{i}-{\hat{y}}_{i}\right)}^{2}}$$

where *y_i_* refers to the observed value, ŷ*_i_* denotes the predicted value, *ȳ* is the mean of the observed values, and n is the total number of observations.

All modeling, evaluation, and visualization processes were conducted in R [28]. The keras, tensorflow, caret, Metrics, and dplyr packages were used for model development and evaluation, while ggplot2 was employed for data visualization. A fixed random seed (set.seed(42)) was applied to ensure reproducibility.

## RESULTS AND DISCUSSION

**Figure 2:**
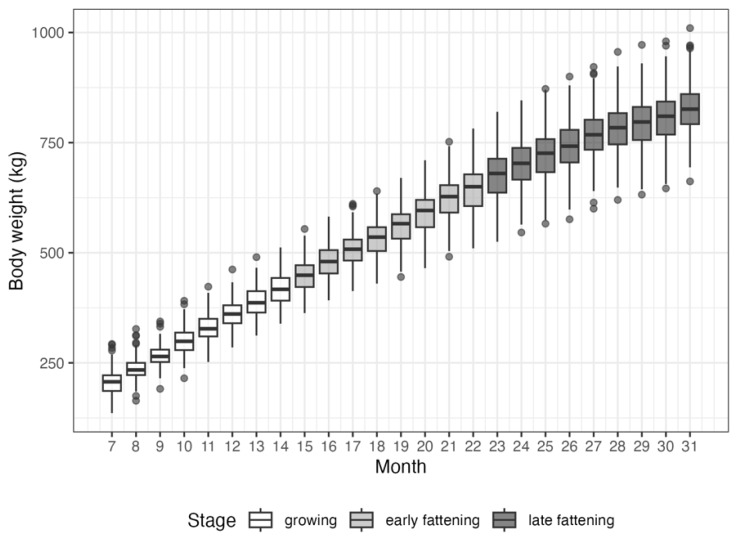
Monthly distribution of body weight (BW) in Hanwoo steers aged 7 to 31 months, classified by growth stage: growing (7–14 months), early fattening (15–22 months), and late fattening (23–31 months).

Figure 2 illustrates the monthly distribution of BW in Hanwoo steers from 7 to 31 months of age, classified into three growth stages. As steers aged, both average and individual differences in BW markedly increased. At 7 months of age (beginning of growing stage), the average BW was 206.3 kg, which was accompanied by a SD of 27.7 kg. It increased to 417.5±34.6 kg by 17 months with greater variation among individuals. The mean BW reached 646.3±52.4 kg at 22 months and 828.5±60.0 kg at 31 months (minimum, 662 kg; maximum, 1,010 kg). These results showed increasing BW variation across animals in later stages. In particular, the observed variation in the late-stage BW reflects high individual variability, which presents a significant challenge for accurately forecasting the final BW. Greater variation among individuals implies distinct growth trajectories, which is influenced by genetic potential, nutritional responses, and environmental interactions [29]. Such individual differences complicate accurate prediction because generalized models may fail to accurately represent animals exhibiting atypical growth patterns. A previous study has highlighted that a substantial proportion of BW variation was attributable to individual animal differences rather than solely from environmental factors [29]. This underscores the necessity of incorporating individualized growth profiles for precise forecasting [30]. Therefore, models accommodating the variability through individualized growth trajectory modeling and sequential BW data inputs can enhance predictive accuracy.

**Figure 3:**
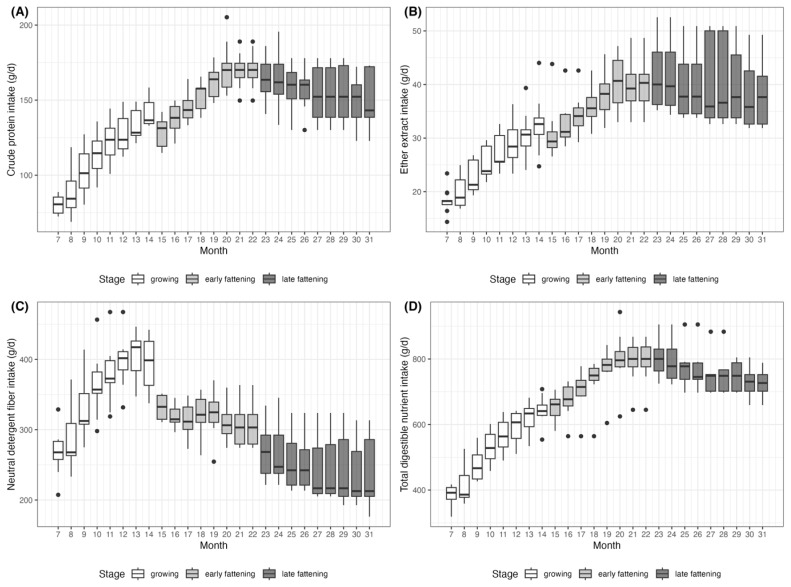
Monthly distribution of nutrient intake for (A) crude protein intake (CPI), (B) ether extract intake (EEI), (C) neutral detergent fiber intake (NDFI), and (D) total digestible nutrient intake (TDNI) across three growth stages in Hanwoo steers: growing (7–14 months), early fattening (15–22 months), and late fattening (23–31 months).

The temporal distribution of nutrient intake from 7 to 31 months of age is shown in Figure 3. The amount of CPI steadily increased from 80.2±5.9 g/day at 7 months to a peak of 172.3±16.2 g/day at 20 months, then decreased to 149.3± 16.0 g/day at 31 months. The interquartile range (IQR) also expanded from 10.7 to 33.7 g/day, indicating increasing variation in CPI. A similar pattern was observed for EEI, which peaked at 40.2±4.3 g/day at 20 months and decreased afterward. NDFI displayed an earlier peak with 400.9±33.1 g/day at 12 months and decreased to 237.3±45.3 g/day by 31 months. Notably, the IQR for NDFI widened dramatically from 25.9 to 80.8 g/day in the late fattening stage. Similarly, TDNI increased from 385.8±24.9 g/day at 7 months to 807.6± 74.8 g/day at 20 months, and then slightly declined to 728.1± 40.1 g/day at 31 months.

These findings support the interpretation that differences between individual animals become more pronounced in the late fattening stage. In Hanwoo production systems, high concentrate diets are typically given during the late fattening stage to increase intramuscular fat content in carcass [31]. However, feeding high concentrate diets during the late fattening stage in Hanwoo steers could reduce feed intake because of possible occurrence of subacute ruminal acidosis caused by rapid starch fermentation and subsequent declines in rumen pH [32,33]. The feed intake variation in the later stages found in the present study apparently resulted from intake reduction in the animals.

Table 4 presents the predictive performance of four models: KNN, XGBoost, RF, and LSTM. The models were evaluated with six different input configurations (I1–I6) representing prediction time points from 17 to 1 month(s) before slaughter. The predictive accuracy was improved as the prediction point approached slaughter in all the models. Among the models, LSTM consistently outperformed others, with R^2^ values improving from 0.60 (I1) to 0.97 (I6) and RMSE values decreasing from 52.80 kg to 22.62 kg in the test set. The highest prediction accuracy was observed for I6 in LSTM. The RF model achieved the highest prediction accuracy at I6 among the ML models, second only to the LSTM, and showed robust generalization. XGBoost exhibited lower predictive accuracy compared with RF across all input configurations, and signs of overfitting were observed from I3 to I6. The lowest predictive accuracy was shown in KNN with its best performance reaching only R^2^ = 0.75 and RMSE = 56.55 kg at I6, which reflected weakest generalization.

The superior performance of the LSTM model appears to stem from its ability to capture sequential dependencies and temporal dynamics, enabling effective use of extended historical sequences [24]. This capability was especially beneficial in the late fattening stage, where increased variability in BW and nutrient intake makes forecasting more difficult. In the present study, the effectiveness of complex models like LSTM was highly dependent on their ability to align with the structure of the input data, and these findings underscore the importance of both input sequence length and appropriate model selection in enhancing prediction accuracy for growth in Hanwoo steers. Forecasting the final BW in advance offers practical advantages to farm-level decision-making including slaughter timing and feeding program adjustment [12]. While the use of longer input sequences can be advantageous for achieving the highest accuracy, this presents a practical compromise for on-farm management. For example, predictions made one month before slaughter (I6) may give a limited time for management such as dietary intervention in farms. Instead, mid-range predictions of I4 (6 months) and I5 (3 months) would allow more time for farm management with relatively high prediction accuracy (R^2^ values of 0.83 and 0.90 and RMSEs of 35.84 kg and 33.12 kg, respectively). Among ML models, the robust generalization of the RF model was indicated by its minor discrepancies between the training and test sets. In contrast, XGBoost showed a tendency toward overfitting as its performance was higher in the training set compared to the test set, and this was more apparent with longer data sequences. The overfitting occurs when a model learns the noise in the training data as well as the underlying patterns and consequently reduces the ability to generalize to new data. This indicates that boosting methods such as XGBoost may be more prone to noise and weaker generalization compared to bagging methods such as RF that provided more stable and consistent performance in the present study [34]. High variation and noise in BW and nutrient intake in our dataset may have favored the variance-reduction strategy of RF over the boosting approach of XGBoost. The overfitting is a significant issue in model development because it hinders the ability of the model to make reliable predictions on unseen data. This can lead to inaccurate management decisions under practical farm conditions. Meanwhile, the large SDs of KNN in the training meant unstable learning performance. This instability is likely to have contributed to the low predictive accuracy observed in the test set, which ultimately reflected weak generalization.

The previous studies in Hanwoo have focused on the current BW estimation with limited attention to time-ahead prediction. For instance, Jang et al [17] estimated the current BW of Hanwoo cattle using 3D imaging techniques including top-view images captured by time-of-flight and stereo vision cameras. Their multiple linear regression models that incorporated body dimensions and age yielded RMSEs between 51.4 and 62.0 kg. Dang et al [16] applied ML models to predict current BW based on ten body measurements including wither height, body length, and chest girth. They achieved higher predictive accuracy compared to the study of Jang et al [17], with RMSEs ranging from 24.75 to 28.55 kg. Despite these improvements, their reliance on body measurements or specialized 3D imaging systems presented practical limitations for real-farm adoption due to cost, labor, and infrastructure burdens [2,35]. In contrast, the present study demonstrated a more accessible and cost-effective approach by utilizing historical data collected from a farm without the need for additional imaging devices or specialized measurement equipment. This highlights the practical advantage of our method for implementation in commercial Hanwoo production systems.

The time-ahead prediction of carcass traits has been explored in studies with different breeds of beef cattle. For instance, Duwalage et al [12] predicted carcass weight in grass-fed beef cattle using seven phenotypic traits: weaning weight, weight gain since weaning, time since weaning, breed, sex, weaning season (wet or dry), and farm property. They developed predictive models using boosted regression trees (a ML method) and multiple linear regression, and reported RMSE values of 11.65, 15.34, 20.76, and 25.06 kg when predicting carcass weight at 1, 3, 9–10 months before slaughter, and at weaning, respectively. Similarly, Alonso et al [36] used zoometric measurements to forecast carcass weight and achieved mean absolute percentage error values of 3.3% and 3.9% at 1 and 3 months, respectively, prior to slaughter. These studies highlighted the feasibility of time-ahead prediction in beef production although their targets (carcass weight) and input features differed from the present study’s live BW and nutrient intake data. The values of RMSEs in the LSTM of the present study were comparable to or slightly higher than the carcass weight prediction RMSEs reported by Duwalage et al [12] and Alonso et al [36]. This is reasonable because live BW is more dynamic and potentially more variable than carcass weight due to temporary factors.

In future studies, the integration of more accurate dry matter intake records and sensor-based BW monitoring would improve both prediction accuracy and scalability [37,38]. Additionally, future research should consider explainability and user-centered design to promote adoption in field settings. This is particularly important because DL models are inherently black-box systems, making it difficult for end users to interpret their internal reasoning processes [39]. Explainable artificial intelligence methods can clarify how specific input features influence predictions, thereby improving model transparency [40]. By integrating these interpretability techniques into decision-support tools, predictive models can become not only more accurate but also more trustworthy and actionable for commercial livestock management.

## CONCLUSION

We developed and compared four predictive models (KNN, XGBoost, RF, and LSTM) to predict the final BW of Hanwoo steers before slaughter using monthly BW and estimated nutrient intake data. Among the tested models, the LSTM model yielded the highest prediction accuracy across all input configurations. When excluding LSTM, RF, XGBoost, and KNN ranked in descending order of performance based on test R^2^ and RMSE across all input configurations. Prediction accuracy improved as the forecasting time point moved closer to the target slaughter age. These models can serve as practical tools to support timely and data-driven decision-making in Hanwoo production systems. In particular, the LSTM model shows strong potential for integration into farm-level decision-support systems and precision feeding programs, which can contribute to more efficient management and optimized resource utilization in commercial Hanwoo production.

## Figures and Tables

**Table 1:** Chemical composition (% DM) of the diets according to the growth stages of Hanwoo steers

Item	Concentrate			Roughage	
	
	Growing^1)^	Early	Late	Timothy	Rice straw
DM	89.40±0.41	88.28±0.37	88.10±0.49	91.98±0.19	85.78±1.90
CP	18.03±0.48	16.01±0.38	15.46±0.70	7.46±1.67	3.89±0.62
EE	4.35±0.89	3.65±0.34	3.78±0.36	1.57±0.18	1.12±0.18
NDF	27.86±4.68	21.75±1.66	20.24±3.11	59.31±2.74	56.11±3.12
TDN	72.33±3.91	72.82±1.59	74.61±2.73	52.72±0.69	44.24±0.36

1) Growth stages were defined as follows: growing (7–14 months), early fattening (15–22 months), and late fattening (23–31 months).

DM, dry matter; CP, crude protein; EE, ether extract; NDF, neutral detergent fiber; TDN, total digestible nutrients.

**Table 2:** Descriptive statistics of the dataset

Item	Mean	SD	Median	Min	Max
CPI (g/d)	141.62	28.30	144.39	25.93	68.91
EEI (g/d)	33.87	8.35	34.36	7.86	14.37
NDFI (g/d)	306.39	63.07	308.82	60.82	176.34
TDNI (g/d)	680.48	132.86	706.24	115.77	318.88
BW (kg)	540.17	197.42	546.00	249.08	136.00

SD, standard deviation; CPI, crude protein intake; EEI, ether extract intake; NDFI, neutral detergent fiber intake; TDNI, total digestible nutrients intake; BW, body weight.

**Table 3:** Summary of six input configurations for predicting final body weight at 31 months of age

Input configuration	Temporal window (mo)	Input length (mo)	Input variables^1)^	Target	Prediction time point (mo before 31 mo)
I1	7–14	8	BW, CPI, EEI, NDFI, TDNI	Final BW	17
I2	7–18	12			13
I3	7–22	16			9
I4	7–25	19			6
I5	7–28	22			3
I6	7–30	24			1

1) All input configurations used five variables.

BW, body weight; CPI, crude protein intake; EEI, ether extract intake; NDFI, neutral detergent fiber intake; TDNI, total digestible nutrient intake.

**Table 4:** Prediction performance of machine and deep learning models with different input configurations

Model	Input configuration^1)^	Training set (CV mean±SD)^2)^		Test set	

		R^2^	RMSE	R^2^	RMSE
KNN	I1	0.39±0.12	47.52±9.66	0.49	68.66
	I2	0.37±0.13	48.78±9.87	0.59	65.28
	I3	0.50±0.29	44.56±10.72	0.56	65.67
	I4	0.50±0.17	43.74±7.16	0.62	62.90
	I5	0.43±0.10	46.80±7.94	0.67	61.37
	I6	0.49±0.19	43.44±13.10	0.75	56.55
XGBoost	I1	0.35±0.13	49.88±5.58	0.54	62.48
	I2	0.58±0.11	39.77±8.32	0.60	59.96
	I3	0.75±0.05	31.38±1.76	0.71	52.39
	I4	0.76±0.10	31.23±4.87	0.76	49.67
	I5	0.84±0.03	24.88±2.05	0.77	44.73
	I6	0.90±0.04	19.51±3.68	0.88	32.16
RF	I1	0.49±0.14	43.74±5.84	0.57	62.45
	I2	0.64±0.09	37.23±5.33	0.67	55.95
	I3	0.76±0.07	30.52±3.23	0.76	50.82
	I4	0.80±0.07	27.51±3.37	0.79	47.75
	I5	0.84±0.07	23.82±5.50	0.85	38.66
	I6	0.90±0.05	19.16±1.37	0.92	29.21
LSTM	I1	0.52±0.15	47.10±9.88	0.60	52.80
	I2	0.74±0.11	40.46±17.87	0.72	45.40
	I3	0.78±0.07	39.19±12.65	0.76	40.92
	I4	0.83±0.09	36.03±12.03	0.83	35.84
	I5	0.90±0.03	32.36±9.28	0.90	33.12
	I6	0.95±0.03	25.45±6.25	0.97	22.62

1) Each input configuration corresponds to sequences ending at 14, 18, 22, 25, 28, or 30 months, which are equivalent to prediction time points of 17, 13, 9, 6, 3, and 1 month(s) before the target age (31 months).

2) Five-fold cross-validation (CV) was performed on the training set (80%), and final model performance was evaluated using a test set (20%).

SD, standard deviation; R^2^, coefficient of determination; RMSE, root mean square error; KNN, k-nearest neighbors; XGBoost, eXtreme Gradient Boosting; RF, random forest; LSTM, long short-term memory.

## Data Availability

Upon reasonable request, the datasets of this study can be available from the corresponding author.
